# Macroscopic hematuria in wasp sting patients: a retrospective study

**DOI:** 10.1080/0886022X.2021.1896547

**Published:** 2021-03-11

**Authors:** Maohe Wang, Singh Prince, Yong Tang, Xiang Zhong, Shasha Chen, Guisen Li, Li Wang, Wei Wang

**Affiliations:** a Zunyi Medical University, Affiliated Hospital of Zunyi Medical University, Zunyi, China; b Department of Nephrology & Institute of Nephrology, Sichuan Academy of Medical Science & Sichuan Provincial People’s Hospital, China; c Suining central hospital, Suining, China; d Division of Nephrology & Hypertension, Mayo Clinic, Rochester, Minnesota, USA

**Keywords:** Wasp sting, macroscopic hematuria, poisoning severity score, AKI, rhabdomyolysis

## Abstract

**Background:**

Macroscopic hematuria after wasp sting has been reported in Asia to occur before acute kidney injury (AKI), and is often used by clinicians as a sign indicating the need for intensive care and blood purification therapy. However, there is no study on the clinical characteristics and prognosis of this symptom.

**Methods:**

The clinical data of 363 patients with wasp sting admitted to Suining Central Hospital from January 2016 to December 2018 were retrospectively analyzed. At admission, the poisoning severity score (PSS) was used as the criterion for severity classification. According to the presence of macroscopic hematuria, the patients were divided into macroscopic hematuria and non-macroscopic hematuria group.

**Results:**

Of the 363 wasp sting patients, 219 were male and 144 were female, with a mean age of 55.9 ± 16.3 years. Fifty-one (14%) had macroscopic hematuria, 39 (10.7%) had AKI, 105 (28.9%) had rhabdomyolysis, 61 (16.8%) had hemolysis, 45 (12.4%) went on to received hemodialysis, and 14 (3.9%) died. The incidence of AKI in macroscopic hematuria group was 70.6%, and oliguric renal failure accounted for 72.2%. Patients with macroscopic hematuria had significantly higher PSS (2.2 ± 0.5 vs. 1.1 ± 0.3, *p* < .001).

**Conclusion:**

Macroscopic hematuria can be regarded as a surrogate marker of deteriorating clinical outcome following wasp stings. In wasp sting patients with symptoms of macroscopic hematuria or serum LDH higher than 463.5 u/L upon admission, the risk of AKI increases significantly, therefore hemodialysis should be considered. The PSS is helpful in early assessment of the severity of wasp sting patients.

## Introduction

Wasps belong to the Order Hymenoptera in the Animal Kingdom [1]. Currently, there are more than 6,000 species of wasps in the world and more than 200 species have been recorded in China [2,3]. Wasp stings are common in the world. Epidemiological surveys in the United States show that wasp stings account for 27.4%–29.7% of all animal injuries and the annual mortality is 0.14–0.74/million population [4,5]. In July–October 2013, 1,675 cases of wasp stings occurred in Shaanxi Province of China, resulting in 42 deaths [6]. However, this global public health problem hasn’t drawn adequate attention as there has no epidemiological survey on wasp stings in China [7]. There is a paucity of existing guidelines on the diagnosis and treatment of wasp stings. Chinese Society of Toxicology has prepared a consensus statement on the standardized diagnosis and treatment of wasp stings [7]. Nevertheless, a wider application of this consensus criteria is likely limited by the complex evaluation criteria. In Europe, the poisoning severity score (PSS) is used to guide initial assessment and appropriate medical care assignment [8–11]. However, the PSS has not been reported specifically for evaluating the severity of wasp sting patients. While local symptoms of wasp sting include redness, swelling, and pain, severe wasp sting can lead to systemic allergic reaction, acute kidney injury (AKI), rhabdomyolysis, hemolysis, shock, and even death [3,12,13]. Xie, etc. [14] reported the occurrence rate of macroscopic hematuria after wasp sting to be 10.1%. Macroscopic hematuria is visible to the naked eye and may appear pink, bright red, brown, or the classic finding of tea colored urine [15,16]. Macroscopic hematuria occurring after wasp sting often alerts clinicians to the likelihood of a serious medical condition which may require intensive care admission, and in some cases even plasmapheresis [7]. However, there has not been any study looking at the clinical characteristics and prognosis of wasp sting patients complicated with macroscopic hematuria in particular. Suining Central Hospital of Sichuan Province, China, is the only tertiary grade A general hospital in the interior areas of Sichuan Province, and most patients with severe wasp stings were treated in our nephrology department and ICU. Therefore, we undertook this study in which we looked at the 363 patients with wasp sting from January 2016 to December 2018 in Suining Central Hospital, and we analyzed the clinical characteristics of patients with macroscopic hematuria. In our study, we used the PSS to evaluate the severity of wasp sting patients upon admission so as to evaluate whether the macroscopic hematuria and the PSS would be valuable to predict the prognosis of severe wasp sting patients.

## Materials and methods

### Research subjects

The research subjects were patients with wasp sting and hospitalized from 1 January 2016 to 31 December 2018 in the nephrology department or the Intensive Care Unit (ICU) of Suining Central Hospital in Sichuan province, China. The exclusion criteria were chronic kidney disease (CKD), age less than 14 years old, and death upon admission. CKD is defined as abnormalities of kidney structure or function, present for >3 months, with implications for health [17]. For the diagnosis of CKD, we did not only rely on the patients’ medical history, but also kidney ultrasonography and baseline serum creatinine with presence of proteinuria/hematuria. AKI was defined by KDIGO definition criteria as any of the following: increase in SCr by ≥ 0.3 mg/dL within 48 h; or increase in SCr ≥1.5 times baseline, which is known or presumed to have occurred within the prior 7 days; or Urine volume < 0.5 mL/kg/h for 6 h [18]. Creatine kinase (CK) levels of 1000 U/L, exceeding five times the upper limit of normal, was used for diagnosing rhabdomyolysis [19]. We categorized them into macroscopic hematuria group (*n* = 51) and non- macroscopic hematuria group (*n* = 312) according to the presence of macroscopic hematuria. Microscopic hematuria was not included in this study. The study was approved by the Ethics Committee of Suining Central Hospital (LLSNCH20200022).

### Data collection

EpiData3.1 software was used to input the following data: 363 patients' demographic indicators, including gender, age, number of stings, the time interval between sting and admission, and previous medical history; main symptoms and signs such as hypotension, allergic rash, macroscopic hematuria, and oliguria (or anuria); the PSS at admission: The PSS grades severity as (0) none, (1) minor, (2) moderate, (3) severe, and (4) fatal poisoning. The severity grading takes into account only the observed clinical symptoms and signs, it does not estimate risks or hazards on the basis of information such as the amounts ingested or serum concentrations of the toxic agent [11]. According to the exclusion criteria, patients with no symptoms (0) and those who died upon admission (4) were excluded, thus the PSS graded the patients into minor, moderate, and severe poisoning levels; laboratory data of patients on admission, the 2nd and the 3rd day after admission, and the day before discharge; severe complications such as AKI, rhabdomyolysis, hemolysis, coagulation disorder, liver dysfunction, MODS, and ARDS; procedures including hemodialysis and plasmapheresis, state at discharge (death or survival), and length of hospital stay.

### Therapeutic schedule

Patients with wasp stings are rarely treated in outpatient clinics. During pre-hospital first aid: given 0.9% sodium chloride to hydration, and glucocorticoid or epinephrine were given for allergic reactions. After admission, the stinger that could be found were removed and the number of stings was counted. 0.9% sodium chloride was given for hydration, and sodium bicarbonate was used to alkalize the urine.

Therapeutic schedule of Rhabdomyolysis: 0.9% sodium chloride was given for hydration, and sodium bicarbonate was used to alkalize the urine. When serum CK was over 1000 U/L, diuretics were given to achieve at least 300 mL/h urine output.

Therapeutic schedule of AKI: Intravenous infusion of glucocorticoid (Methylprednisolone 40 mg/d intravenous infusion) for 3–5 days, and the dosage was gradually reduced and discontinued for 7–10 days. hydration and diuresis for patients without oliguria or anuria, to achieve at least 100–200 mL/h urine excretion.

Blood purification treatment: We provided blood purification therapy when patients with wasp sting had macroscopic hematuria (14%) and if they had AKI (oliguria or KDIGO stage 3), and in some patients with CK over 10000 u/L in the absence of AKI.

Our patients were treated with glucocorticoids (Methylprednisolone 40 mg/d intravenous infusion) for 3–5 days in the presence of macroscopic hematuria, AKI, or anaphylaxis, and the dosage was gradually reduced and discontinued for 7–10 days.

### Statistical methods

The data were analyzed by SPSS software version 19.0. The enumeration data are represented by rate, and the chi-square test was used for comparison between the two groups. The measurement data conforming to the normal distribution are represented by mean ± standard deviation ($\bar{x}$ ± s), and the non-conforming normal distribution by median M (P25, P75). Variables of the two groups were compared by Mann-Whitney U test. Spearman analysis was performed for the correlation between macroscopic hematuria and the patients’ outcome. Multivariate logistic regression model was used to screen the risk factors of AKI, and then ROC curve analysis was performed on the selected risk factors. A *p*-value of less than .05 for 95% confidence was set and used as a cutoff point to examine the statistical association between the variables.

## Results

### Demographic characteristics

**Figure 1.**
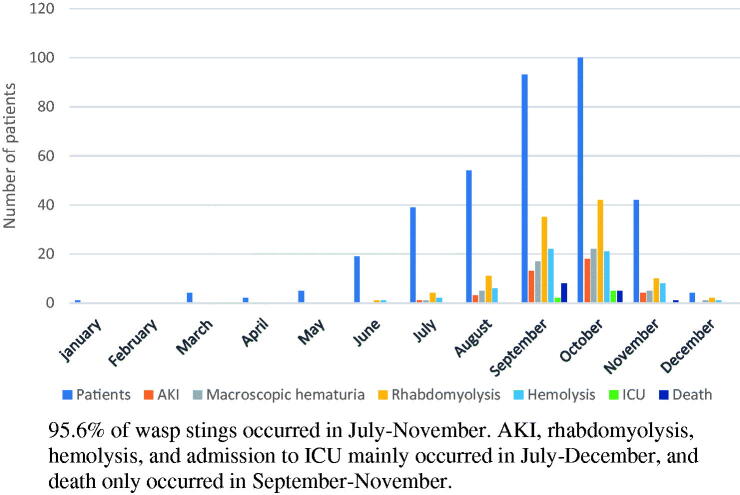
Monthly distribution of patients with severe complication.

From January 2016 to December 2018, 390 patients with wasp sting presented to Suining Central Hospital. Three hundred sixty-three were included in the study. Twenty-four patients refused to be hospitalized and three patients were excluded from the study as they already had CKD based on their medical history and ultrasonography. Sixty percent patients were male with a mean age of 55.9 ± 16.3 years (Table 1). The time interval between sting and admission was 3(0.5–144) hours, and the PSS was 1.2 ± 0.5 points on admission. Fourteen patients died with a mortality of 3.9%. Eight patients died on the first day of admission, followed by three each on second and third day. The most common cause of death was ARDS (9/14) followed by MODS (4/14) (Table 1). Figure 1 shows the monthly distribution of patients with macroscopic hematuria, rhabdomyolysis, hemolysis, AKI, ICU and death after wasp sting.

**Table 1.** Clinical data of wasp sting patients (*n* = 363).

Variable	Value
Age (years)	55.9 ± 16.3
Gender (M: F)	219:144
Number of stings	9 (4.15)
Interval (hours)*	3 (2.5)
Poisoning severity score	1.2 ± 0.5
Hospitalization Days (day)	4 (3.6)
Allergic rash *n* (%)	50 (13.8)
Macroscopic hematuria *n* (%)	51 (14%)
Hypotension *n* (%)	20 (5.5)
AKI *n* (%)	39 (10.7)
AKI stage1	7
AKI stage2	9
AKI stage3	23
Rhabdomyolysis *n* (%)	105 (28.9)
Hemolysis *n* (%)	61 (16.8)
Oliguria or anuria *n* (%)	27 (7.4)
Coagulation abnormalities *n* (%)	139 (38.3)
Liver damage *n* (%)	82 (22.6)
Dialysis *n* (%)	45 (12.4)
MODS** *n* (%)	56 (15.4)
ARDS**^#^***n* (%)	13 (3.6)
ICU**^##^***n* (%)	7 (1.9)
Death *n* (%)	14 (3.9)

*The time interval between sting and admission, **Multiple Organ Dysfunction Syndrome, **^#^**Acute Respiratory Distress Syndrome, **^##^**Intensive Care Unit.

### Clinical manifestations

Almost all patients had local symptoms such as redness, swelling, and pain at the sting site. Thirty-nine patients (10.7%) had AKI, 7(17.9%) patients in stage1, 9 (23.1%) in stage2, 23 (59%) in stage 3. Fifty-one patients (14%) had macroscopic hematuria, 61 patients (16.8%) developed hemolysis, and 105 patients (28.9%) developed rhabdomyolysis. Forty-five patients (12.4%) underwent blood purification therapy, and 7 patients (1.9%) were admitted to ICU. Of the 7 patients admitted to ICU, five were directly admitted to ICU for need of endotracheal intubation, and 2 were transferred from nephrology department (Table 1).

### Comparison of clinical data between the two groups

There was no statistical difference in gender composition between patients with macroscopic hematuria and patients with non-macroscopic hematuria (*p* = .089). The average age of patients with macroscopic hematuria was higher than that of patients with non-macroscopic hematuria (65 years vs 54 years, *p* < .001). The number of stings, the time interval between stings and admission, and the PSS on admission in patients with macroscopic hematuria group were higher than those in patients with non-macroscopic hematuria group, and the hospitalization days were significantly prolonged (*p* < .001). Thirteen patients (25.5%) died in the macroscopic hematuria group, and 1 patient (0.3%) died in the non-macroscopic hematuria group (*p* < .001). The patient in the non-macroscopic hematuria group died of respiratory failure in the setting of chronic obstructive pulmonary disease. Although there was no statistical difference in the duration of blood purification therapy between these two groups, the time of blood purification therapy in the macroscopic hematuria group was significantly prolonged (Table 2).

**Table 2.** Clinical data between non-macroscopic hematuria and macroscopic hematuria group.

Variable	non-macroscopic hematuria group (*n* = 312)	macroscopic hematuria group (*n* = 51)	*p*
Age (years)	54.4 ± 16.7	65.2 ± 9.5	<.001
Gender (M: F)	194:118	25:26	.089
Number of stings	7 (3.10)	30 (19.33)	<.001
Interval (hours)*	3 (2.4)	6 (4.12)	<.001
Poisoning severity score	1.1 ± 0.3	2.2 ± 0.5	<.001
Hospitalization Days (day)	4 (3.6)	11 (3.21)	<.001
Allergic rash *n* (%)	50 (16)	0 (0)	.001
Hypotension *n* (%)	19 (6.1)	1 (2)	.332
AKI *n* (%)	3 (1)	36 (70.6)	<.001
AKI stage1	0	7	
AKI stage2	1	8	
AKI stage3	2	21	
Rhabdomyolysis *n* (%)	55 (17.6)	50 (98)	<.001
Hemolysis *n* (%)	15 (4.8)	46 (90.2)	<.001
Oliguria or anuria *n* (%)	1 (0.3)	26 (51)	<.001
Coagulation abnormalities *n* (%)	90 (28.8)	49 (96.1)	<.001
Liver damage *n* (%)	35 (11.2)	47 (92.2)	<.001
Dialysis *n* (%)	5 (1.6)	40 (78.4)	<.001
Dialysis time (day)	3 (2.5, 5.5)	7 (2, 19.7)	.268
MODS** *n* (%)	8 (2.6)	48 (94.1)	<.001
ARDS**^#^***n* (%)	0 (0)	13 (25.5)	<.001
ICU**^##^***n* (%)	0 (0)	7 (13.7)	<.001
Death *n* (%)	1 (0.3)	13 (25.5)	<.001

*The time interval between sting and admission, **Multiple Organ Dysfunction Syndrome, **^#^**Acute Respiratory Distress Syndrome, **^##^**Intensive Care Unit.

### Comparison of complications between the two groups

No allergic rash was seen in the macroscopic hematuria group (*p* = .001). One patient developed hypotension in the macroscopic hematuria group compared to 19 in the non-macroscopic hematuria group (*p* = .332). The incidence of oliguria (or anuria), rhabdomyolysis, hemolysis, coagulation abnormalities, liver damage, MODS, ARDS in the macroscopic hematuria group was higher than that in the non-macroscopic hematuria group. Seven patients (13.7%) in the macroscopic hematuria group were admitted to ICU, while no patients in the non-macroscopic hematuria group were admitted to ICU (*p* < .001). Thirty-six (70.6%) patients with macroscopic hematuria developed AKI, compared to 3 (1%) patients with non-macroscopic hematuria (*p* < .001). Forty patients (78.4%) in the macroscopic hematuria group received hemodialysis compared to 5 (1.6%) in the non-macroscopic hematuria group (*p* < .001) (Table 2).

### Comparison of laboratory examination results between the two groups

At the time of admission and the 2nd–3rd day after admission, the serum creatinine, creatine kinase (CK), aspartate aminotransferase (AST), indirect bilirubin (IBIL), alanine transaminase (ALT), prothrombin time (PT), activated partial thromboplastin time (APTT), lactate dehydrogenase (LDH), leukocyte (WBC) values in the macroscopic hematuria group were higher than those in the non-macroscopic hematuria group (*p* < .001). Serum creatinine and LDH tested before discharge were still significantly higher in the macroscopic hematuria group than that in the non-macroscopic hematuria group (*p* < .001) (Table 3).

**Table 3.** Lab examination between non-macroscopic hematuria and macroscopic hematuria group.

Variable		non-macroscopic hematuria group (*n* = 312)	macroscopic hematuria group (*n* = 51)	*p*
Creatinine	On admission	0.7 (0.6, 0.9)	1.0 (0.8, 1.8)	<.001
(0.7–1.2mg/dL)	The 2nd–3rd day	0.8 (0.6, 0.9)	1.7 (0.8, 3.5)	<.001
	Pre-discharge	0.7 (0.6, 0.8)	1.7 (0.8, 2.3)	<.001
Creatine kinase	On admission	174 (108.5, 318)	1785.5 (565, 5581.7)	<.001
(40–200U/L)	The 2nd–3rd day	516 (142.2, 1525)	5408.5 (2446.7, 18127.5)	<.001
	Pre-discharge	83 (46, 285)	91.5 (38.2, 260)	.603
AST*	On admission	33 (26, 45)	419 (168, 776.7)	<.001
(13–35U/L)	The 2nd–3rd day	41 (27, 72.5)	441 (197, 903)	<.001
	Pre-discharge	28 (20.5, 45)	24 (18, 44)	.332
Indirect bilirubin	On admission	0.5 (0.3, 0.7)	2.3 (1.5, 3.6)	<.001
(0–1.0mg/dL)	The 2nd–3rd day	0.2 (0.2, 0.4)	1.3 (0.8, 2.4)	<.001
	Pre-discharge	0.3 (0.2, 0.4)	0.2 (0.2, 0.6)	.947
ALT**	On admission	21 (16, 31)	104 (46.7, 288)	<.001
(7–40U/L)	The 2nd–3rd day	33 (22, 53)	138.5 (50.7, 390.2)	<.001
	Pre-discharge	32.5 (22, 55)	28 (15, 92)	.576
PT**^#^**	On admission	13.6 (13, 14.5)	14.7 (13.6, 15.7)	<.001
(11–14.5s)	The 2nd–3rd day	13 (12.4, 13.7)	14.3 (13.4, 15.2)	<.001
	Pre-discharge	12.7 (12.1, 13.2)	13.6 (12.1, 14.2)	.091
APTT**^##^**	On admission	46.2 (36.2, 85.9)	111.9 (84.2, 176.8)	<.001
(26–40s)	The 2nd–3rd day	34 (31, 38)	60.2 (36.9, 176.8)	<.001
	Pre-discharge	32.9 (29.6, 36.1)	37.5 (33.1, 40.4)	.003
LDH	On admission	206 (177, 248)	1211 (868, 1945)	<.001
(120–250U/L)	The 2nd–3rd day	212 (164.2, 301.5)	1462 (838.7, 2182)	<.001
	Pre-discharge	184.5 (156.2, 248.7)	475 (299, 637)	<.001
Leukocyte	On admission	11 (8, 14.9)	21.9 (15.5, 27.5)	<.001
(3.5–9.5*10^9^/L)	The 2nd–3rd day	10.9 (8.8, 13.6)	17.3 (13.9, 23.2)	<.001
	Pre-discharge	8.8 (6.9, 10.3)	9.1 (6.6, 13.9)	.306
Hemoglobin (130–175g/L)	On admission	140 (126, 151)	129 (112, 140)	<.001
	The 2nd–3rd day	126 (113, 139)	103 (85, 115)	<.001
	Pre-discharge	124 (112, 143)	81 (74, 94)	<.001
Platelet (125–350*10^9^/L)	On admission	171 (126, 215)	201 (120, 251)	.096
	The 2nd–3rd day	167 (131, 206)	95 (63, 123)	<.001
	Pre-discharge	182 (139, 223)	178 (123, 218)	.548
Calcium (2.08–2.8mmol/L)	On admission	2.25 (2.13, 2.36)	2.08 (1.88, 2.24)	<.001
	The 2nd–3rd day	2.09 (1.99, 2.29)	1.95 (1.85, 2.12)	<.001
	Pre-discharge	2.17 (2.08, 2.27)	1.95 (1.79, 2.12)	<.001
Urea (2.86–8.2mmol/L)	On admission	5.53 (4.75, 7.03)	8.58 (7.05, 13.6)	<.001
	The 2nd–3rd day	5.88 (4.72, 7.93)	9.88 (7.48, 19.00)	<.001
	Pre-discharge	6.17 (5.06, 7.18)	8.14 (6.09, 13.21)	<.001
Potassium (3.5–5.3mmol/L)	On admission	3.43 (3.21, 3.77)	4.0 (3.51, 4.36)	<.001
	The 2nd–3rd day	3.05 (2.78, 3.44)	3.69 (2.99, 4.14)	<.001
	Pre-discharge	3.09 (2.70, 3.52)	3.56 (2.96, 3.82)	.022

*Aspartate aminotransferase, **Alanine transaminase, **^#^**Prothrombin time, **^##^**Activated partial thromboplastin time.

### Spearman analysis between macroscopic hematuria and patients' outcome

A Spearman correlation analysis indicated that macroscopic hematuria was related to the patients' outcome (*r* = 0.454, *p* < .001) (Table 4).

**Table 4.** Spearman analysis between macroscopic hematuria and the patients’ outcome.

	Outcome		Summation
	Survival	Death	
Macroscopic hematuria			
No	311	1	312
Yes	38	13	51
Summation	349	14	363

Spearman analysis, *r* = 0.454 *p* < .001.

### Multivariate logistic regression analysis

The PSS was one risk factor for death in wasp sting patients (OR = 6.768, 95%CI 1.981–23.120, *p* = .002) (Table 5).

**Table 5.** Multivariate logistic regression analysis of death in wasp sting patients.

Variable	β	Wald χ2	*p*	OR	95%CI
Poisoning severity score	1.912	9.306	.002	6.768	1.981–23.120

H-L test X² = 0.826, *p* = .999.

We took the data of laboratory examination with significant difference at admission (AKI group and non-AKI group) (Table 6) as independent variables and performed a multivariate logistic regression analysis of AKI in wasp sting patients. The results showed that LDH was an independent risk factor for AKI (OR = 1.006, 95%CI 1.004–1.007, *p* < .001) (Table 7).

**Table 6.** Lab examination on admission between non-AKI and AKI group.

Variable	non-AKI group (*n* = 324)	AKI group (*n* = 39)	*p*
Creatine kinase (40–200U/L)	181.5 (110, 327.75)	1841 (568, 5542.5)	<.001
AST* (13–35U/L)	33 (26, 49)	448.5 (160.75, 1149.25)	<.001
ALT** (7–40U/L)	21 (16, 32)	104 (50.75, 556.25)	<.001
Indirect bilirubin (0–1.0mg/dL)	0.45 (0.30, 0.79)	2.83 (1.56, 3.98)	<.001
LDH (120–250U/L)	207 (178, 254.5)	1562 (832.5, 2089)	<.001
Leukocyte (3.5–9.5 × 10^9^/L)	11.3 (8.1, 15.42)	21.9 (15.6, 28.1)	<.001

*Aspartate aminotransferase, **Alanine transaminase.

**Table 7.** Multivariate logistic regression analysis of AKI in wasp sting patients.

Variable	β	Wald χ2	*p*	OR	95%CI
LDH	0.006	46.709	<.001	1.006	1.004–1.007

H-L test X² = 14.555, *p* = .068.

### ROC curve analysis

**Figure 2.**
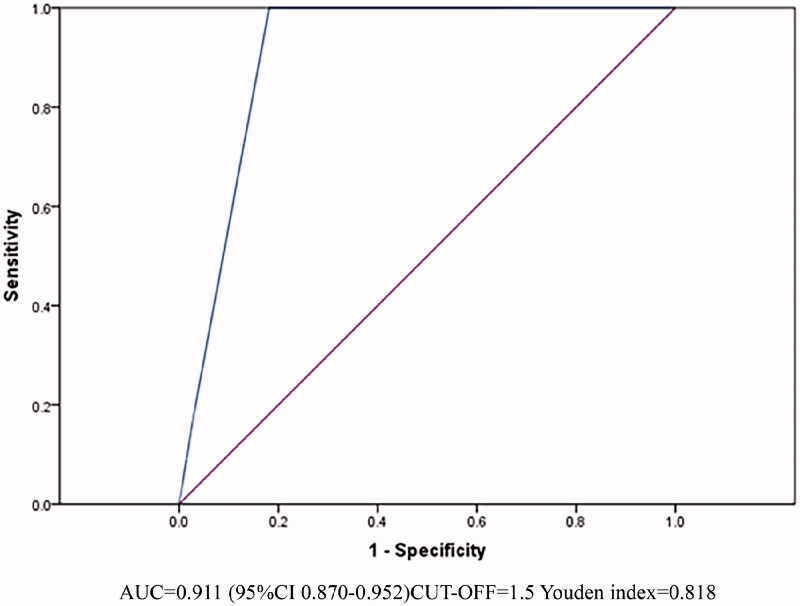
The poisoning severity score on admission to predict death of wasp sting patients.

The AUC of the PSS on admission to predict death of wasp sting patients was 0.911(95%CI 0.870–0.952, *p* < .001) (Figure 2).

**Figure 3.**
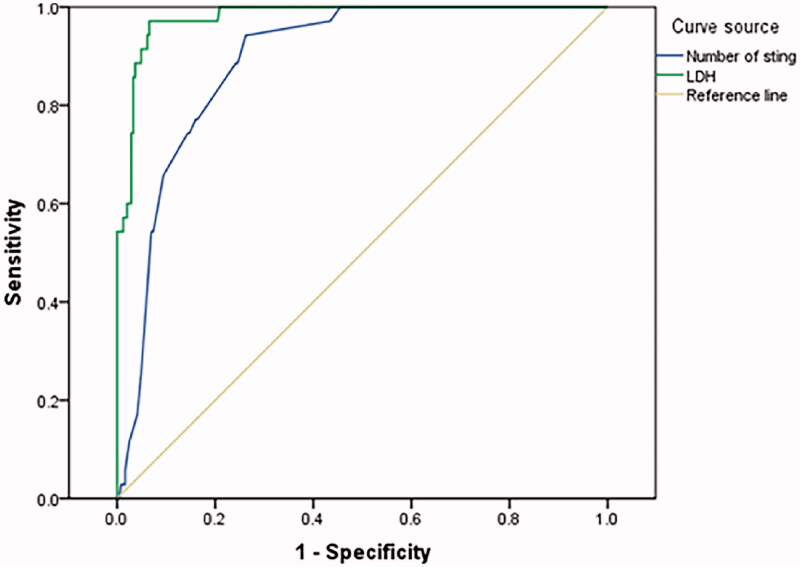
LDH on admission and number of stings to predict AKI of wasp sting patients. LDH on admission AUC = 0.980(95%CI 0.966–0.995), CUTOFF = 463.5u/L Youden index = 0.906 Number of stings AUC = 0.875(95%CI 0.826–0.925), CUTOFF = 11.5, Youden index = 0.663.

The AUC of serum LDH on admission to predict AKI of wasp sting patients was 0.980(95%CI 0.966–0.995, *p* < .001) (Figure 3).

The AUC of number of stings to predict AKI of wasp sting patients was 0.875(95%CI 0.826–0.925, *p* < .001) (Figure 3).

## Discussion

The current study is a retrospective analysis showing clinical features of patients with wasp sting in Suining Central Hospital, Sichuan, China. Macroscopic hematuria after wasp sting is not rare in Asian countries [6,14,20]. Our study shows that 14% of the patients with wasp sting had macroscopic hematuria, and this was only seen in the summer and fall months from July through December. More than half of patients with macroscopic hematuria (51%) developed oliguria (or anuria). More than 70% of patients with macroscopic hematuria had AKI, and the mortality was 25.5%. The poisoning severity score (PSS) in patients with macroscopic hematuria was significantly higher than that in patients with non-macroscopic hematuria (2.2 ± 0.5 vs. 1.1 ± 0.3, *p* < .001). The PSS was one risk factor for death in wasp sting patients.

Wasp venom contains a variety of bioactive components, such as enzymes (including phospholipase, hyaluronic acid), amines (including histamine, serotonin, catecholamine), peptides (including wasp venom peptide, wasp kinin) [12]. Phospholipase damages the cell membrane by attacking the phospholipid structure, which has a toxic effect on skeletal muscle and erythrocyte membrane, leading to rhabdomyolysis and intravascular hemolysis [21–23]. Wasp venom peptide can also independently cause muscle necrosis and cell apoptosis [12,24]. Venom induced rhabdomyolysis and intravascular hemolysis leads to a release of muscle enzymes such as creatine kinase and muscle protein such as myoglobin, free hemoglobin (from red blood cells) in the intravascular circulation [25]. Once in the circulation, these muscle (heme) proteins gets freely filtered through the glomeruli and eventually exceed the tubular reabsorption capacity of renal tubules resulting in macroscopic hematuria [16]. Kidney biopsy study in wasp sting-induced kidney injury has demonstrated deposition of myoglobin and hemoglobin in renal tubules [26–28].

In developed countries, wasp stings are mainly manifested in varying degrees of allergic-reactions, therefore their treatment mainly focuses on desensitization and antiallergic treatment [5,29,30]. On the other hand, in China, wasp sting patients are mainly characterized by more severe and life-threatening presentations such as MODS, ARDS and non-allergic shock [13,14], which was also shown in our cohort. Population based data from United States and Sweden shows that wasp stings mostly occur in summer and autumn months when the climate is warm and hence there is more outdoor activity and thus subsequent an increased exposure to wasps [31,32]. In our study as well, 95.6% of wasp stings occurred in the months of July through November. Severe complications such as AKI, rhabdomyolysis, hemolysis, and admission to ICU mainly occurred in July-December, but death was documented to only happen in September-November months. This regional and seasonal difference may be related to the different wasp species in different regions and seasons, and the different components and virulence of wasp venom [21,33,34].

Prior study has shown that macroscopic hematuria associated with wasp sting generally occurs 4–12 h after sting, and tends to occur earlier than AKI [35]. In our cohort, 14% of wasp sting patients presented with macroscopic hematuria, and the incidence of serious complications such as AKI, MODS, and ARDS of these patients were significantly higher than those reported by Xie, etc.[14]. Patients admitted to ICU only occurred in the macroscopic hematuria group, and almost 92.8% of the death were from the macroscopic hematuria group. Spearman analysis also demonstrated that macroscopic hematuria was related to patients' outcome (*r* = 0.454, *p* < .001). Thus, we suggest that macroscopic hematuria may be regarded as a surrogate marker of worse clinical outcomes following wasp stings. These patients also had a higher PSS at the time of admission. PSS is already used in Europe to assess the severity of poisoned patients (including environmental toxins), with simple and accurate characteristics [9,36]. Multivariate logistic regression analysis showed that the PSS was also an independent risk factor for death in wasp sting patients (OR = 6.768, 95%CI 1.981–23.120, *p* = .002). The ROC analysis of the PSS for predicting death in wasp sting patients shows that when the PSS over 1.5, the risk of death was increased with high accuracy (AUC = 0.911). In brief, the PSS is helpful in early assessment of the severity of wasp sting patients and is worthy of promotion in clinical practice.

The serum creatine kinase, aspartate aminotransferase, lactate dehydrogenase, and indirect bilirubin of patients with macroscopic hematuria were significantly higher than those of patients with non-macroscopic hematuria on admission and the 2nd–3rd day after admission, which indicated that patients with macroscopic hematuria experienced more severe and prolonged rhabdomyolysis and intravascular hemolysis. Prior studies have suggested that rhabdomyolysis, intravascular hemolysis, and direct nephrotoxicity of wasp venoms are the main causes of AKI in patients with wasp stings [37–41], and 77–90% of the pathology were ATN [42,43]. The kidney injury is exacerbated in low volume states which accompany severe wasp sting due to development of shock [16,44]. AKI is the most common and serious outcome following wasp sting [12,45,46]. Ten-point seven percent of our patients developed AKI, 82% of patients with AKI had stages 2/3 of kidney injury by KDIGO criteria. The incidence of oliguric AKI was as high as 72.2% in macroscopic hematuria group, and most of them required blood purification treatment. In Xie's literature, the overall incidence of AKI was higher in the group with more than 10 stings [14]. The ROC analysis of our patients showed that AKI incidence increased when the stings was higher than 11 (AUC = 0.875, 95%CI 0.826–0.925, *p* < .001). Among biochemical parameters, serum LDH upon admission was found to be an independent marker for AKI. Zhang, etc. has shown that elevated serum LDH was associated with AKI in wasp sting patient population [13,35]. ROC analysis of LDH for predicting AKI in wasp sting patients shows in our study that when serum LDH was over 463.5 U/L, the risk of AKI in wasp sting patients increased, with high accuracy (AUC = 0.980, 95%CI 0.966–0.995, *p* < .001). Thus, greater number of sting and elevated LDH on admission (> 463.5 u/L) are associated with greater risk of AKI and therefore should alert clinicians of more serious outcomes such as need of hemodialysis.

In the macroscopic hematuria group, the serum creatinine of 25 patients complicated with AKI did not return to normal at discharge (1.3–10.5 mg/dL). Only 8 patients were followed up in outpatient for 2–8 months, and renal function was not fully recovered in 4 patients (creatinine 1.28–2.65 mg/dL). According to Zhang's report, 10.7% of patients with wasp sting complicated with AKI will progress to CKD [13]. We have evidence from population based studies that a subset of patients with AKI progress to CKD [47]. It would be therefore be advisable for such patients to be followed in nephrology clinic after their discharge. However, this result may also be related to the short hospitalization time of our patients (average 11 days), because, according to Ambarsari's report, the cure of acute kidney injury after wasp sting takes 3–6 weeks [42].

Our research also has limitations. Ours is a retrospective study, hence there may be selection bias in addition to possible confounding. The study data came from a single center and we did not have complete information such as wasp species, sting site, prognosis and follow-up of patients with AKI. We did not consider the possible effect of microscopic hematuria, and we also didn't distinguish between hemoglobinuria and myoglobinuria. Although the PSS has a high accuracy in predicting the prognosis of wasp sting patients, the severity of wasp sting patients from our study highlighted that the number of stings, time of the year also correlate with worse outcomes. PSS criteria may benefit with incorporation of these variables. Twelve patients had diabetes, but no evidence of diabetic nephropathy was found. Our study did not adjust for risk of AKI with preexisting diabetes. To our knowledge, this is the largest study in terms of study subject number to review macroscopic hematuria in wasp sting patients in terms of clinical features and outcomes.

In conclusion, macroscopic hematuria is one of the early and important markers of adverse outcomes in patients with wasp sting, and thus, can alert the clinicians to initiate prompt and careful monitoring of such patients. In wasp sting patients with symptoms of macroscopic hematuria or serum LDH higher than 463.5 u/L upon admission, the risk of AKI increases significantly, therefore hemodialysis should be considered. The poisoning severity score is helpful in early assessment of the severity of wasp sting patients.

## Data Availability

All data and material were obtained from Suining Central Hospital.

## References

[1] Pesek RD, Lockey RF. Management of insect sting hypersensitivity: an update. Allergy Asthma Immunol Res. 2013;5(3):129–137. 23638310 10.4168/aair.2013.5.3.129 PMC3636446

[2] Walker AA, Robinson SD, Yeates DK, et al. Entomo-venomics: the evolution, biology and biochemistry of insect venoms. Toxicon. 2018; 154:15–27. 30267720 10.1016/j.toxicon.2018.09.004

[3] Yang X, Chai L, Liu C, et al. Serum metabolomics analysis in wasp sting patients. Biomed Res Int. 2018;2018:1–8. 10.1155/2018/5631372 PMC6323449 30671459

[4] Forrester JA, Weiser TG, Forrester JD. An update on fatalities due to venomous and nonvenomous animals in the United States (2008–2015). Wilderness Environ Med. 2018;29(1):36–44. 29373216 10.1016/j.wem.2017.10.004

[5] Forrester JA, Holstege CP, Forrester JD. Fatalities from venomous and nonvenomous animals in the United States (1999–2007). Wilderness Environ Med. 2012;23(2):146–152. 22656661 10.1016/j.wem.2012.02.012

[6] Liu Z, Li XD, Guo BH, et al. Acute interstitial nephritis, toxic hepatitis and toxic myocarditis following multiple Asian giant hornet stings in Shaanxi Province, China. Environ Health Prev Med. 2016;21(4):231–236. 26910407 10.1007/s12199-016-0516-4 PMC4907929

[7] , Hubei Provincial P, Occupational Disease U, Yang X, et al. Expert consensus statement on standardized diagnosis and treatment of wasp sting in China. Zhonghua Wei Zhong Bing Ji Jiu Yi Xue. 2018;30(9):819–823. 30309405 10.3760/cma.j.issn.2095-4352.2018.09.001

[8] Casey PB, Dexter EM, Michell J, et al. The prospective value of the IPCS/EC/EAPCCT poisoning severity score in cases of poisoning. J Toxicol Clin Toxicol. 1998;36(3):215–217. 9656976 10.3109/15563659809028941

[9] Cairns R, Buckley NA. The poisoning severity score: if it did not exist, we would have to invent it. J Med Toxicol. 2017;13(2):131–134. 28516408 10.1007/s13181-017-0614-8 PMC5440327

[10] Mong R, Arciaga GJ, Tan HH. Use of a 23-hour emergency department observation unit for the management of patients with toxic exposures. Emerg Med J. 2017;34(11):755–760. 28768699 10.1136/emermed-2016-206531

[11] Persson HE, Sjoberg GK, Haines JA, et al. Poisoning severity score. Grading of acute poisoning. J Toxicol Clin Toxicol. 1998;36(3):205–213. 9656975 10.3109/15563659809028940

[12] Gong J, Yuan H, Gao Z, et al. Wasp venom and acute kidney injury: the mechanisms and therapeutic role of renal replacement therapy. Toxicon. 2019;163:1–7. 30880185 10.1016/j.toxicon.2019.03.008

[13] Zhang L, Yang Y, Tang Y, et al. Recovery from AKI following multiple wasp stings: a case series. Clin J Am Soc Nephrol. 2013;8(11):1850–1856. 24009218 10.2215/CJN.12081112 PMC3817916

[14] Xie C, Xu S, Ding F, et al. Clinical features of severe wasp sting patients with dominantly toxic reaction: analysis of 1091 cases. PLoS One. 2013;8(12):e83164. 24391743 10.1371/journal.pone.0083164 PMC3877022

[15] Sandhu KS, LaCombe JA, Fleischmann N, et al. Gross and microscopic hematuria: guidelines for obstetricians and gynecologists. Obstet Gynecol Surv. 2009;64(1):39–49. 19099611 10.1097/OGX.0b013e3181932841

[16] Bagley WH, Yang H, Shah KH. Rhabdomyolysis. Intern Emerg Med. 2007;2(3):210–218. 17909702 10.1007/s11739-007-0060-8

[17] KDIGO 2012 clinical practice guideline for the evaluation and management of chronic kidney disease. Kidney Int Suppl. 2013;3:1–150. 10.1038/ki.2013.243 23989362

[18] Section 2: AKI Definition. Kidney Int Suppl. 2012;2(1):19–36. 10.1038/kisup.2011.32 PMC4089595 25018918

[19] Michelsen J, Cordtz J, Liboriussen L, et al. Prevention of rhabdomyolysis-induced acute kidney injury – a DASAIM/DSIT clinical practice guideline. Acta Anaesthesiol Scand. 2019;63(5):576–586. 30644084 10.1111/aas.13308

[20] Sigdel MR, Raut KB. Wasp bite in a referral hospital in Nepal. J Nepal Health Res Counc. 2013;11(25):244–250. 24908524

[21] Habermann E. Bee and wasp venoms. Science. 1972;177(4046):314–322. 4113805 10.1126/science.177.4046.314

[22] Fernandez ML, Quartino PY, Arce-Bejarano R, et al. Intravascular hemolysis induced by phospholipases A2 from the venom of the Eastern coral snake, Micrurus fulvius: functional profiles of hemolytic and non-hemolytic isoforms. Toxicol Lett. 2018;286:39–47. 29197624 10.1016/j.toxlet.2017.11.037

[23] Perez-Riverol A, Lasa AM, Dos Santos-Pinto JRA, et al. Insect venom phospholipases A1 and A2: roles in the envenoming process and allergy. Insect Biochem Mol Biol. 2019;105:10–24. 30582958 10.1016/j.ibmb.2018.12.011

[24] Konno K, Kazuma K, Nihei K-I. Peptide toxins in solitary wasp venoms. Toxins. 2016;8(4):114. 27096870 10.3390/toxins8040114 PMC4848640

[25] Silva GBDJ, Vasconcelos AGJ, Rocha AMT, et al. Acute kidney injury complicating bee stings – a review. Rev Inst Med Trop Sao Paulo. 2017;59:e25. 28591253 10.1590/S1678-9946201759025 PMC5459532

[26] Chao YW, Yang AH, Ng YY, Yang WC. Acute interstitial nephritis and pigmented tubulopathy in a patient after wasp stings. Am J Kidney Dis. 2004;43(2):e15-19. 14750120 10.1053/j.ajkd.2003.10.025

[27] Dhanapriya J, Dineshkumar T, Sakthirajan R, et al. Wasp sting-induced acute kidney injury. Clin Kidney J. 2016;9(2):201–204. 26985369 10.1093/ckj/sfw004 PMC4792632

[28] Kularatne K, Kannangare T, Jayasena A, et al. Fatal acute pulmonary oedema and acute renal failure following multiple wasp/hornet (*Vespa affinis*) stings in Sri Lanka: two case reports. J Med Case Rep. 2014;8:188. 24929921 10.1186/1752-1947-8-188 PMC4088920

[29] Welton RE, Williams DJ, Liew D. Injury trends from envenoming in Australia, 2000–2013. Intern Med J. 2017;47(2):170–176. 27749012 10.1111/imj.13297

[30] Warrell DA. Venomous bites, stings, and poisoning: an update. Infect Dis Clin North Am. 2019;33(1):17–38. 30712761 10.1016/j.idc.2018.10.001

[31] Mowry JB, Spyker DA, Brooks DE, et al. 2014 Annual Report of the American Association of Poison Control Centers’ National Poison Data System (NPDS): 32nd annual report. Clin Toxicol. 2015;53(10):962–1147. 10.3109/15563650.2015.1102927 26624241

[32] Johansson B, Eriksson A, Ornehult L. Human fatalities caused by wasp and bee stings in Sweden. Int J Legal Med. 1991;104(2):99–103. 2054310 10.1007/BF01626039

[33] Vikrant S, Jaryal A, Gupta D, et al. Epidemiology and outcome of acute kidney injury due to venomous animals from a subtropical region of India. Clin Toxicol. 2019;57(4):240–245. 10.1080/15563650.2018.1513526 30306815

[34] Burdmann EA, Jha V. The authors reply. Kidney Int. 2017;92(5):1288–1289. 10.1016/j.kint.2017.08.007 29055429

[35] Li F, Liu L, Guo X, et al. Elevated cytokine levels associated with acute kidney injury due to wasp sting. Eur Cytokine Netw. 2019;30(1):34–38. 31074416 10.1684/ecn.2019.0425

[36] Wang IJ, Yeom SR, Park SW, et al. Poison severity score and sequential organ failure assessment score: carbon monoxide poisoning prognosis. PLoS One. 2019;14(3):e0212025. 30822313 10.1371/journal.pone.0212025 PMC6396897

[37] Grisotto LS, Mendes GE, Castro I, et al. Mechanisms of bee venom-induced acute renal failure. Toxicon. 2006;48(1):44–54. 16774771 10.1016/j.toxicon.2006.04.016

[38] Sakthirajan R, Dhanapriya J, Varghese A, et al. A: clinical profile and outcome of pigment-induced nephropathy. Clin Kidney J. 2018;11(3):348–352. 29942498 10.1093/ckj/sfx121 PMC6007272

[39] Basnayake K, Cockwell P, Hutchison CA. Rhabdomyolysis and acute kidney injury. N Engl J Med. 2009;361(14):1411–1412. 19797292 10.1056/NEJMc091538

[40] Dvanajscak Z, Walker PD, Cossey LN, et al. Hemolysis-associated hemoglobin cast nephropathy results from a range of clinicopathologic disorders. Kidney Int. 2019;96(6):1400–1407. 31668630 10.1016/j.kint.2019.08.026

[41] Ling Z, Yi T, Fang L, et al. Multiple organ dysfunction syndrome due to massive wasp stings: an autopsy case report. Chin Med J. 2012;125(11):2070–2. 22884081

[42] Ambarsari CG, Sindih RM, Saraswati M, et al. Delayed admission and management of pediatric acute kidney injury and multiple organ dysfunction syndrome in children with multiple wasp stings: a case series. Case Rep Nephrol Dial. 2019;9(3):137–148. 31828077 10.1159/000504043 PMC6902257

[43] Vikrant S, Parashar A. Acute kidney injury due to multiple Hymenoptera stings-a clinicopathological study. Clin Kidney J. 2017;10(4):532–538. 29340149 10.1093/ckj/sfx010 PMC5761506

[44] Zager RA. Rhabdomyolysis and myohemoglobinuric acute renal failure. Kidney Int. 1996;49(2):314–326. 8821813 10.1038/ki.1996.48

[45] Vikrant S, Parashar A. Wasp venom-induced acute kidney injury: a serious health hazard. Kidney Int. 2017; 92(5):1288. 10.1016/j.kint.2017.05.035 29055428

[46] Yang L. Acute kidney injury in Asia. Kidney Dis. 2016;2(3):95–102. 10.1159/000441887 PMC5123008 27921036

[47] Devarajan P, Jefferies JL. Progression of chronic kidney disease after acute kidney injury. Prog Pediatr Cardiol. 2016;41:33–40. 27429539 10.1016/j.ppedcard.2015.12.006 PMC4943846

